# Prostate cancer treatment with Irreversible Electroporation (IRE): Safety, efficacy and clinical experience in 471 treatments

**DOI:** 10.1371/journal.pone.0215093

**Published:** 2019-04-15

**Authors:** E. Guenther, N. Klein, S. Zapf, S. Weil, C. Schlosser, B. Rubinsky, M. K. Stehling

**Affiliations:** 1 Vitus Prostate Center, Institut für Bildgebende Diagnostik, Offenbach, Germany; 2 Department of Information and Communication Technologies, Universitat Pompeu Fabra, C/Roc Boronat, Barcelona, Spain; 3 Department of Mech. Engineering, University of California Berkeley, Berkeley, CA, United States of America; University of South Alabama Mitchell Cancer Institute, UNITED STATES

## Abstract

**Background:**

Irreversible Electroporation (IRE) is a novel image-guided tissue ablation technology that induces cell death via very short but strong pulsed electric fields. IRE has been shown to have preserving properties towards vessels and nerves and the extracellular matrix. This makes IRE an ideal candidate to treat prostate cancer (PCa) where other treatment modalities frequently unselectively destroy surrounding structures inducing severe side effects like incontinence or impotence. We report the retrospective assessment of 471 IRE treatments in 429 patients of all grades and stages of PCa with 6-year maximum follow-up time.

**Material and findings:**

The patient cohort consisted of low (25), intermediate (88) and high-risk cancers (312). All had multi-parametric magnetic resonance imaging, and 199 men had additional 3D-mapping biopsy for diagnostic work-up prior to IRE. Patients were treated either focally (123), sub-whole-gland (154), whole-gland (134) or for recurrent disease (63) after previous radical prostatectomy, radiation therapy, etc. Adverse effects were mild (19.7%), moderate (3.7%) and severe (1.4%), never life-threatening. Urinary continence was preserved in all cases. IRE-induced erectile dysfunction persisted in 3% of the evaluated cases 12 months post treatment. Mean transient IIEF-5-Score reduction was 33% within 12-month post IRE follow-up and 15% after 12 months. Recurrences within the follow-up period occurred in 10% of the treated men, 23 in or adjacent to the treatment field and 18 outside the treatment field (residuals). Including residuals for worst case analysis, Kaplan Maier estimation on recurrence rate at 5 years resulted in 5.6% (CI95: 1.8–16.93) for Gleason 6, 14.6% (CI95: 8.8–23.7) for Gleason 7 and 39.5% (CI95: 23.5–61.4) for Gleason 8–10.

**Conclusion:**

The results indicate comparable efficacy of IRE to standard radical prostatectomy in terms of 5-year recurrence rates and better preservation of urogenital function, proving the safety and suitability of IRE for PCa treatment. The data also shows that IRE, besides focal therapy of early PCa, can also be used for whole-gland ablations, in patients with recurrent PCa, and as a problem-solver for local tumor control in T4-cancers not amenable to surgery and radiation therapy anymore.

## Introduction

The incidence of prostate cancer (PCa) is increasing worldwide, with an estimated 1.1 million new cases in 2012 [1]. Established PCa-treatments like radical prostatectomy (RPE) or radiation therapy (RT) aim to treat the whole prostate, which is associated with side effects such as impotence, incontinence and potential damage to the rectum and bladder [2][3], whilst at best providing only moderate survival benefits compared to active surveillance [4]. It has recently been shown that the largest PCa lesion with the highest tumor grade predominantly influences the progression of disease [5][6], suggesting that treatment of this index lesion alone would be highly effective [7][8]. Focal thermal therapies such as Cryosurgery, Radiofrequency Ablation (RFA) or High Intensity Focal Ultrasound (HIFU) have gained ground as minimally invasive alternatives to established therapies [9][10]. In comparison, Irreversible Electroporation (IRE), a novel tissue ablation technology, has the advantage of being non-thermal with elements of tissue selectivity [11], which significantly reduces toxicity on vital anatomical structures surrounding the malignancy. Because of these properties, IRE might become a standard therapy for PCa, and further, a problem solver for advanced T4-cancers for which there are few other treatment options and/or only treatable with resulting permanent damage.

To date, six studies with smaller patient cohorts have been published on IRE of PCa [12–17]. However, no larger case series were published to the best of our knowledge. There is an ongoing multi-center register trial (NCT02255890). This publication presents the retrospective evaluation of 471 IRE treatments, discussing feasibility, safety, toxicity, functional and oncological outcomes.

## Patients and methods

### Patient selection and ethical approval statement

Men with prostate cancer who would potentially benefit from IRE-treatment of their PCa and who refused all types of standard therapy were included, with the primary goal of significant tumor mass reduction, and complete local tumor ablation if achievable. Patients with all stages of disease were included. Patients unfit for total intravenous anesthesia and patients with defibrillators were excluded because there was no statement from the manufactures regarding the safety in conjunction with pulsed electrical field treatments.

All patients underwent meticulous informed consent about the nature of their disease, the prognosis, the established treatment options according to the S3-guidelines for the treatment of PCa issued by the German Urological Society, the technique and experimental nature of the IRE treatment and the details of the diagnostic work-up concerning the localization of cancer foci in their prostate. As the data analysis is retrospective, a formal consent specific to the data analyzed, summarized and presented here, is not required. Each treatment was personalized according to the patient’s individual needs and wishes (individual medical treatment). Data collection was purely retrospective. No treatment was adapted to suit scientific purposes.

### Patient cohort

Between May 2011 and October 2016 471 tissue ablation procedures in 429 patients with PCa were performed, using IRE (NanoKnife, AngioDynamics Inc., USA). Table 1 summarizes patient demographics, cancer grade and stage. Seventy patients had had PCa related treatments prior to IRE: Sixteen patients had undergone radical prostatectomy (RPE), twenty-three had had radiation therapy (RT), 5 had had both. Seventeen patients had undergone a transurethral resection of the prostate (TURP), 8 had been treated with High-Intensity Focused Ultrasound (HIFU), two of which had had the procedure performed twice. The majority (N = 29) of those with previous treatments had undergone androgen deprivation therapy (ADT).

**Table 1:** Patient demographics, cancer stage and grade. Most patients had intermediate to high risk cancers and a Gleason score of 7a (159) or 7b (66). Whilst organ-confined cancers predominated (T1a –T2c, N = 313), 115 cases were non-organ confined (T3a –T4, approx. 27%). In 9 patients no Gleason score was available, as they had refused biopsy. In four patients d’Amico risk classification was impossible due to lack of biopsy, whereas in five patients a PSA exceeding 20 ng/ml or clinical stage of ≥ T2c resulted in a high-risk classification.

Patient demographics		Cancer stage and grade									
Mean age /years	64 ± 8	*D’Amico Risk Classification*						*Gleason Score*		*TNM Staging*	
Mean V of prostate /ml	32 ± 18	N/A				4		3+3	82	T1a-T1c	32
Mean PSA /ng/ml	10 ± 250	Low				25		3+4 / 4+3	225	T2a-T2c	281
# Patients with biopsy	420	Intermediate				88		4+4	68	T3a-T3b	54
# Patients with prior treatment*		High				312		5+3 / 3+5	3	T4 and any with N1 and/or M1	61
Total no. of patients	429	D’Amico High Risk Group Sub-classes:						> 4+4	42	N/A	1
			“+1”	“+2”	“+3”		N/A	N/A	9		
		PSA	133	86	81		12				
		Gleason	48	100	164		0				
		Stage	10	20	281		1				

*prior treatments are further explained in the text.

### Diagnostic work-up

All patients underwent multi-parametric Magnetic Resonance Imaging (mpMRI) at least once prior to IRE [18], except one patient with a cardiac pace maker. Apart from nine, all patients (N = 420) had histo-pathological confirmation of PCa (Table 1). Almost half of the patients (N = 199) underwent 3D-mapping biopsy, either as the initial type of biopsy or secondarily to determine the spacial distribution of PCa in their prostate; the technique employed was similar to the one described in the paper of Onik et al. [19] and is further described in S1 Text.

### Procedures

The IRE electrodes (AngioDynamics Inc., USA) were manually inserted through the perineum under ultrasound guidance without a brachytherapy grid, as previously described [20]. The IRE-field was planned in a way that it exceeded the macroscopic tumor extent by at least 8mm towards the center of the prostate, often more, when the additional ablation volume associated with this was deemed not to cause any additional adverse effects and by increasing the distance between electrode pairs provide a more advantageous, larger ablation field. Towards the capsule the electrodes were, whenever possible, placed within a couple millimeters inside the prostatic capsule. The treatment field geometry was estimated primarily using the treatment field estimator provided by the IRE generator (NanoKnife, Angiodynamics, European version) and results from finite element simulations as described in detail in [21].

Trans-rectal Ultrasound (US) was used for real-time guidance, together with MRI and often PSMA-PET/CT (Prostate Specific Membrane Antigen-Positron Emission Tomography/Computated Tomography) for orientation via cognitive fusion. US/MRI fusion systems were tested but discarded early on because they are inaccurate due to distortion of the prostate during the IRE procedure, which prevents accurate image fusion. All treatments were carried out with the NanoKnife (AngioDynamics Inc., USA). All treatment parameters are summarized in Table 2A and 2B. All treatments were carried out within 24 hours with an overnight stay of the patients.

**Table 2:** A. IRE treatment parameters of the presented patient cohort. B. Ablation field extent. C. Procedure categories of patients with more than one IRE application.

**A. IRE Parameters**	
Total number of procedures	471
Total number of patients	429
Mean number of electrodes used	5 ± 1
Mean procedure time	120 ± 34 min
Mean set voltage-to-distance-ratio	1518.13 ± 204.05 V/cm
Mean ablated ablated /%*	72.4 ± 25.9%
**B. Treatment Extent**	
Uni-lobar or focal (≤50%)	123
Bi-lobar but not whole gland (>50% and ≤90%)	153
Whole gland (>90%)	134
Cannot be determined or recurrence	63
**C. Re-treatments categorized**	
Sum of re-treatments	43
Tumor extent/prostate-size related	8
Incomplete ablation/tumor residue	3
In-field recurrence	12
Out-of-field recurrence	16
Extra prostatic cancer (i.e. seminal vesicles)	3

*according to MRI evaluation 24h post IRE.

### Categorization of re-treatments

Of the 471 IRE-treatments, 43 were IRE-re-treatments after previous IRE (Table 2C). In this section, the rationales for the re-treatments are explained and categorized.

#### 1. Tumor extent/prostate-size related

IRE ablation volume is limited by 3 factors: maximal length of 1.5cm of the electrically conducting tip (“exposed length”) of the electrode, maximal distance between electrodes of 2 cm, and the total number of electrodes used, which for most but a few exceptions we limited to 7, because more are very difficult to handle in the restricted space of the perineum. The limited exposed length requires the electrodes to be re-positioned from apex to base in larger ablations (“push-forward”); this severely limits visibility on US by gas formation from electrolysis [20] such that more than one push-forward is almost impossible. Thus, in very large cancers, which could not be completely ablated with a max. of 7 electrodes and one push-forward, the ablation was split into two IRE procedures.

#### 2. Incomplete ablation/tumor residue

In this case, tumor residues were discernible immediately after the IRE-procedure and were removed during a second IRE procedure.

#### 3. In-field recurrence

In this case, there was no discernible tumor mass immediately after IRE but tumor re-growth 3 months or later in or adjacent to the IRE-ablation field. These recurrences most likely occurred because the IRE-field did not encompass the whole extent of the tumor.

#### 4. Out-of-field recurrence

This type of "recurrence" occurred outside the IRE-ablation field, in untreated prostatic tissue. In the proper sense, these were diagnostic failures, i.e. missed macroscopic tumor foci due to sub-optimal diagnostic work-up (some patients refused re-MRI and re-biopsy). Recurrent PCa outside a 1 cm range around the treatment field (i.e. outside the border of the ablation zone) were considered as out-of-field failures.

#### 5. Extra prostatic cancer

Patients with infiltrated seminal vesicles who had IRE treatment were included in this patient cohort.

### Follow-up

Inclusion cut-off date (date of IRE) and follow-up cut-off time for follow-up data inclusion were determined such that a minimum follow-up time of 4 months resulted. Routine follow-up comprised PSA-tests and MRI scans [21]. Whilst PSA-testing was recommended every 3 months in the first 2 years, then every 6m, MRI was recommended after 1 day, at 3, 6, 12 months post IRE, then annually. Biochemical recurrences were defined by a rise in PSA above the baseline value at 3 months post IRE with confirmation by multi-parametric MRI, in some cases by additional biopsy or PSMA-PET/CT. The matrix shown in Fig B in S1 Text summarizes the resulting acquired data points. Additionally, patients were interviewed concerning adverse events and their evolution over time. For long-term assessment of toxicity and functional and oncological outcome, patients were grouped according to their maximum follow-up time in order to have an objective result of the treatment progress (Table 3 and Fig A in S1 Text). Follow-up included mpMRI and/or PSA levels, as described in detail in S1 Text.

**Table 3:** A. Follow-up period of all treated patients. B. Number of patients who completed the IIEF-5 Questionnaire. C. Number of patients who gave subjective feedback on their sexual functions. D. Number of patients who completed the IPSS-Questionnaire.

A. Follow-up Period		B. IIEF-5-Questionnaire		C. Subjective Assessment of ED		D. IPSS-Questionnaire	
N total included	429	N total (with pre IRE score)	294	N total included	361	N total included	260
N at least one data point	385	N pre and post IRE with initial score > 7	155			N valid and complete input with pre- and at least one post- IRE score	155
N at least one follow-up within 6m	344	N without adjuvant therapies (i.e. HDT)	140				
N at least one follow-up between 6m and 12m	244	N all of the above and valid/complete input (included)	124			N valid input on quality of life score pre- and at least one post-IRE	125
N over 12m follow up	171	Median time to first/last follow-up	129/ 288			Median time to first/last follow-up	101/ 241
N last data point ≤ 12m	258						

### Assessment of safety

Acute toxicity was recorded intra- and post-operatively for all treatments until the time of removal of the Foley catheter. All patients had an MRI 24h post IRE to confirm the congruence of the IRE-field with the extent of the tumor, and to assess potential procedure-related side effects, e.g. hemorrhage and rectal damage.

### Assessment of genitourinary function

Urinary continence was primarily assessed by interviewing the patients concerning any involuntary loss of urine related to the IRE-treatment and the different forms of incontinence (i.e. stress-, urge, overflow-incontinence). If they affirmed incontinence, they were followed-up more frequently, until the symptoms subsided. The International Prostate Symptom Score (IPSS) was included in the evaluation process of toxicity and outcome after IRE treatment of PCa, as it contains questions to urinary symptoms and one question concerning quality of life. Table 3C summarizes the number of patients who completed the evaluation process. In addition to the International Consultation on Incontinence Questionnaire-Urinary Incontinence (ICIQ-UI) assessments, continence status pre and post treatment and during the entire follow-up period was assessed.

Erectile function (ED) was evaluated by two methods. Firstly, by the standard IIEF-5-Score before and after IRE. Secondly, by our own evaluation algorithm, in which we asked patients whether they 1) had experienced any negative change in erectile function related to IRE and 2) were unable to have satisfactory intercourse (use of PDE-5 inhibitors was allowed, when requested by the patient) and no spontaneous nocturnal erection. Patients in whom both 1 and 2 applied were classified as having an IRE-related significant ED. Table 3B and 3C summarizes the number of patients who successfully completed the evaluation process.

### Assessment of oncological outcome / recurrence free survival (RFS)

To measure oncological outcome, MRI and PSA were the primary and routine indicators for the incident of recurrence, Choline or PSMA-PET/CT and re-biopsy were additionally acquired when either PSA or MRI indicated a suspicious lesion (PI-RADS ≥ 4). The PSA criterium for recurrence were 3 consecutive PSA increases (ASTRO definition) [22], as the Nadir required for the PHOENIX definition [23] was found difficult to assess after IRE, because fluctuating and often decreasing PSA levels were observed in some cases even more than 9 months after IRE in larger ablation zones. The MRI criterium for recurrence were in accordance with the criteria defined in PI-RADS v1 (until 2016) and v2 (2016 onwards) [24]. The morphological changes, diffusion coefficient, T2 and DCE changes were in accordance with the findings recently published by Scheltema et al. [21]. All cases who were classified as potential recurrences according to the modalities outlined above or by other external sources that showed suspicion or confirmation of a recurrence. All data was discussed by a board of urologists and/or oncologists and radiologists who had at least 10 years of experience in the field. Conservatively, biochemical recurrences were noted in cases where at least two strong indications, one very strong indication or histological confirmation existed. The patient was then recommended to undergo re-biopsy and, if positive for cancer, re-treatment if this was considered the best choice for the patient.

### Statistical analysis

Statistical analysis was performed using scripts written in Wolfram Research [https://www.wolfram.com/mathematica/] Mathematica. The Wilcoxon-Mann-Whitney-Test was used to assess statistically significant differences in paired continuous variables (all questionnaire outcomes) and unpaired continuous variables (e.g. parameters concerning prostate volume and involvement of neurovascular bundles), respectively. Analyses of regions of interest from imaging were performed by means of OsiriX MD [https://www.osirix-viewer.com/osirix/osirix-md/] and Merge eFilm Workstation [https://estore.merge.com/na/estore/content.aspx?productID=444]. Statistical significance was set at p < 0.05. Kaplan-Meier curves and analysis of oncological outcome was performed with Prism GraphPad 5 [https://www.graphpad.com/scientific-software/prism/], with a confidence interval of 95%.

## Results

### Feasibility of the procedure

Tumor ablation with IRE was primarily successful in all treatments except three, which were classified as in-field failures. Treatment field sizes are listed in Table 2B. In cases of IRE of recurrent PCa, treatment can possibly be hampered to varying degrees by scarring, calcifications and distortions of the prostate, impeding US visibility and electrode insertion. IRE re-treatments were only minimally hampered by anatomical distortion and poorer ultrasound visibility.

### Toxicity

Relatively few adverse events occurred, mostly only mild to moderate (Table 4). Transient urinary retention and/or dysuria were amongst the most common, both more frequent in large prostates and/or large ablation zones. Permanent urinary retention occurred in 4 patients, requiring TURP to restore normal urination. One of the first patients in this series developed a recto-prostatic fistula (closing spontaneously after a few weeks), one patient experienced a bladder perforation by a faulty catheter, and one patient suffered from prolonged urogenital-tract infection, resulting in a testicular abscess which required orchidectomy; this infection was not directly IRE-related since it occurred approx. 10 days after IRE after multiple complicated catheter manipulations at an outside clinic, and was therefore excluded from the statistics on continence and potency evaluation entirely but kept in the statistics on adverse events.

**Table 4:** Adverse events after IRE for PCa.

Mild	19.7%
Mild hematuria	3.8%
Transient urinary retention	9.1%
Dysuria	6.8%
**Moderate**	**3.7%**
Prostatitis	0.2%
Proctitis (uncertain genesis)	0.2%
Epididymiditis	0.6%
Pseudo post vasectomy syndrome	0.2%
Urinary tract infection	2.5%
**Severe or medically significant**	**1.4%**
Permanent urinary retention	0.8%
Recto-prostatic fistula	0.2%
Bladder perforation by catheter	0.2%
Severe prostatitis	0.2%
**Life-threatening consequences**	**0%**
**Death related to adverse events**	**0%**

### Urinary continence

In patients fully continent before IRE, no urinary incontinence was observed 12 months post IRE or later during the observation period. Assessment was based on electrode position and 24h post-procedure MRI, which showed edema and lack of contrast agent uptake. IPSS-Score analysis revealed that in 7.7% of the evaluated patients scores increased temporarily from below 8 to above 19 (severe symptoms) after IRE (Fig C in S1 Text). In one case, the last included data point still showed a score above 20. In terms of urinary symptoms, 72.8% of evaluated patients reported no change or an improvement in quality-of-life, 27.2% a decrease. To the date of data acquisition cut-off, the last available data point still shows dissatisfaction (≥ 5 points) in one patient who was initially satisfied (≤ 2 points).

### Erectile function

Erectile function (EF) before and after IRE was evaluated in 124 patients with standard IIEF-5 questionnaires. Fourteen men (11.3%) developed severe erectile dysfunction (ED: IIEF5 >7 before IRE and IIEF5 ≤7 after IRE), which persisted for longer than one year in four men (3%).

**Fig 1:**
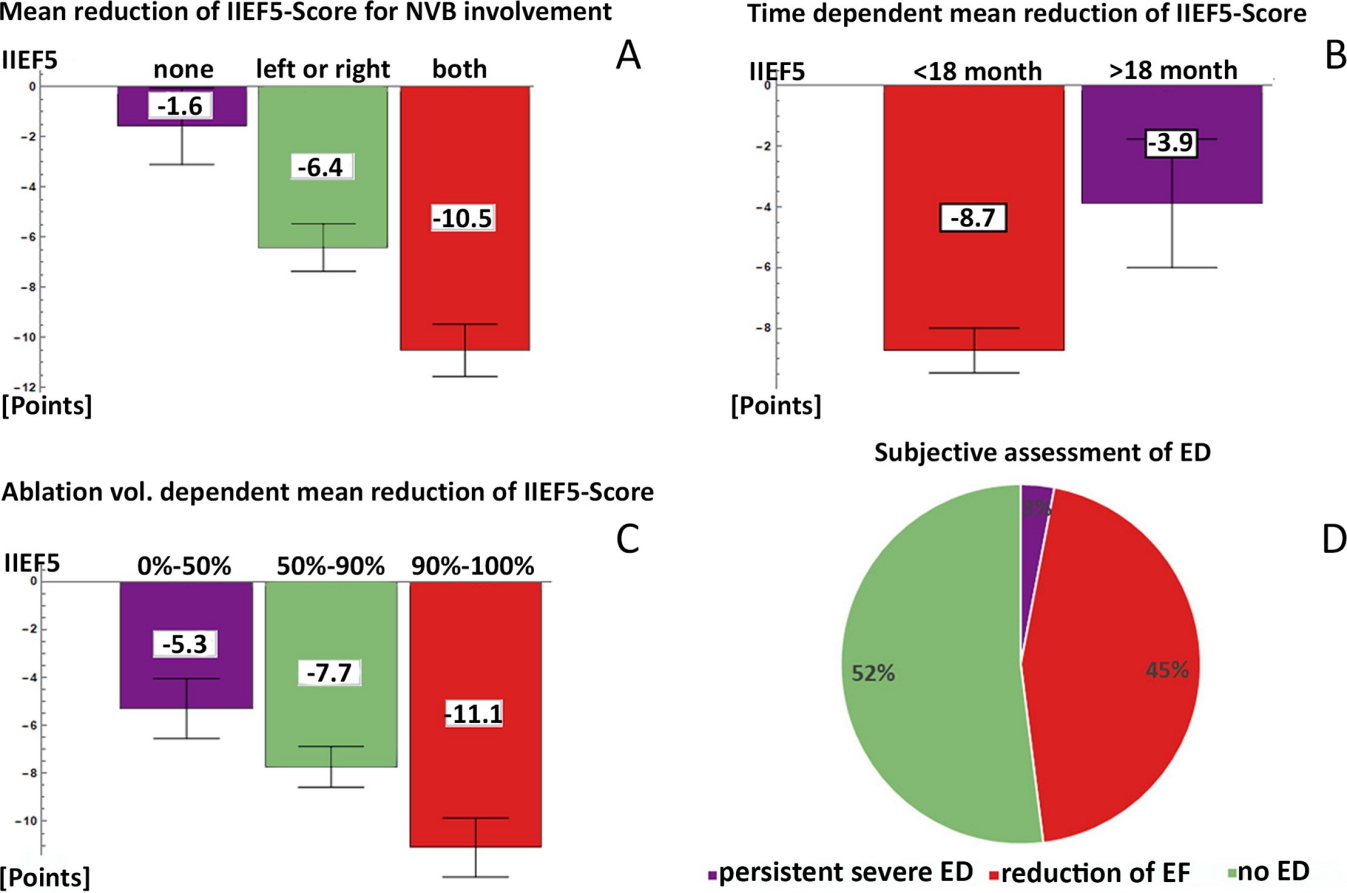
Erectile function after IRE for PCa. A. Correlation between IIEF5-Score reduction with the ablation volume: less than 50% of the prostate treated (left, purple, -5.3 points), 50 to 90% of prostate volume ablated (middle, green, -7.73 points) and whole gland ablation (right, red, -11.1 points). B. Correlation between IIEF5-Score reduction and extent of neurovascular bundle (NVB) involvement in the IRE-field. The columns show the most recently obtained mean IIEF5-Score (where approx. 2/3 were within the first year after IRE): no NVB (left, purple, -1.57 points), one (left or right) NVB (middle, green, -6.42 points) and both NVB (right, red, -10.52 points). C. Reduction of the IIEF5-Score less than 18 months (left, red, -8.72 points) and more than one year after IRE (right, purple, -3.88 points). D. Outcome of subjective assessment of erectile dysfunction (ED). 52% had no ED, 45% experienced transient ED (occurrence of reduction of ED within 1 year after IRE), and 3% experienced persistent IRE-induced ED.

Fig 1 shows the correlations of the IIEF-5-Score with ablation volume (1A), neurovascular bundle (NVB) involvement (1B) and time post IRE (1C).

Fig 1A shows that smaller ablation volumes (<50% of the prostate) resulted in a mean IIEF-5-Score reduction of 17.7% (-5.3 points), in whole-gland ablations (100% ablation volume) it was twice as high (37%, -11.1 points). Fig 1B shows that NVB involvement also correlates positively with ED; assessment of NVB involvement in the treatment field was based on the absence of contrast agent uptake on 24h post-IRE MRI. Fig 1C shows a statistically significant improvement (p = 0.045) of EF over time (>18m post IRE): from a mean reduction of the IIEF-5-Score of 33% (-8.72 points) during the first year after IRE, to 15% (-3.88 points) after 18 months. Thus, data shows a recovery of EF after IRE.

Subjective assessment revealed no reduction of EF in 52% and transient reduction of EF in approximately 45% of all IRE treated patients (Fig 1D).

### Efficacy–recurrence free survival

Oncological efficacy of IRE was determined by local tumor control or recurrence-free survival (RFS). Recurrent PCa was determined by a rise in PSA value with corresponding findings on mpMRI and/or PSMA-PET/CT (prostate specific membrane antigen positron emission tomography). For details see supplement materials.

**Fig 2:**
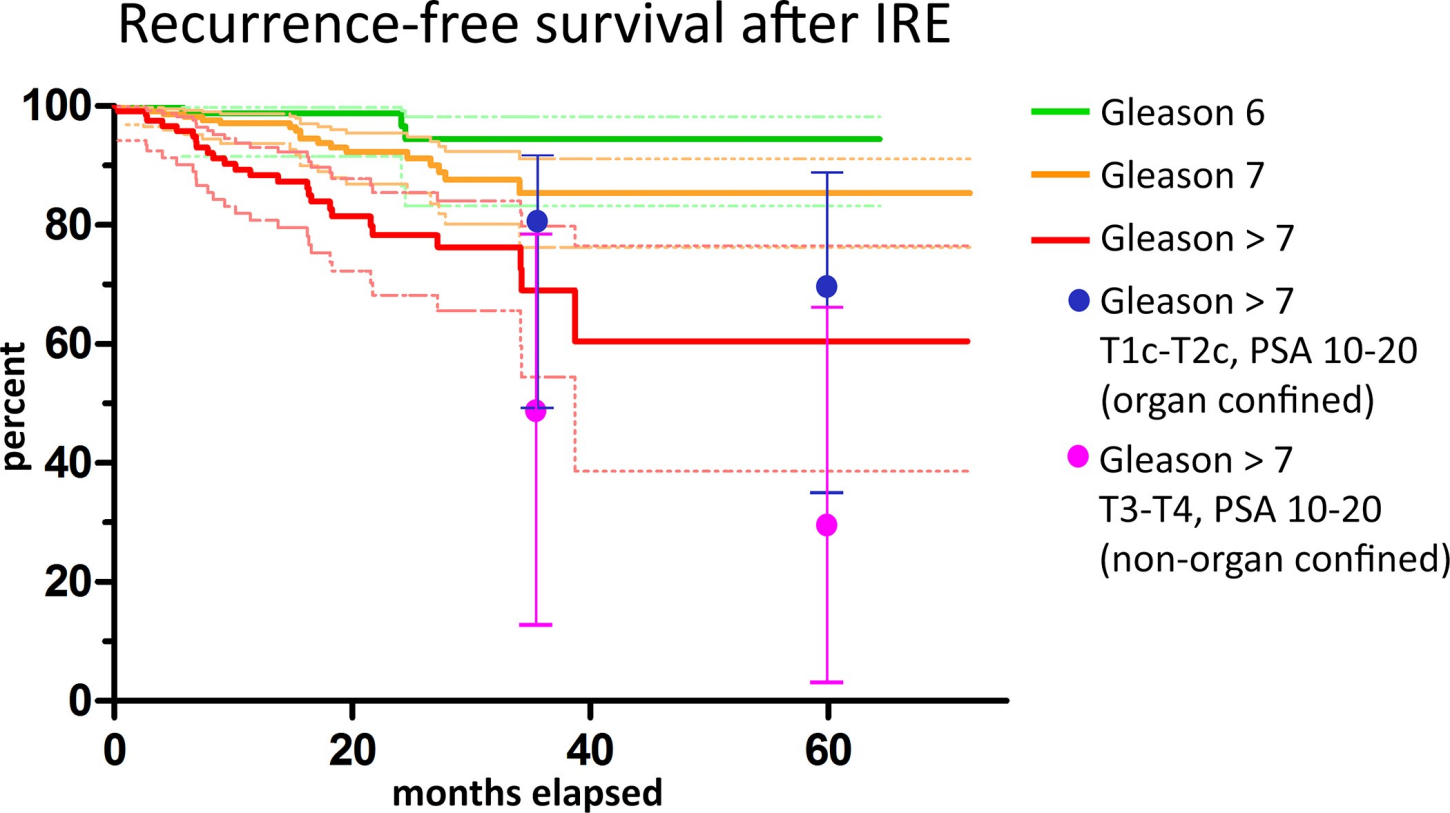
Kaplan-Meier curves of recurrence free survival for PCa treated with IRE at 72 months follow-up; Gleason 6 (green), Gleason 7 (orange) and Gleason >7 (red), with 95% CI (dashed thin lines). Recurrence-free survival rates were: Gleason 6: 94%, Gleason 7: 85%, Gleason >7: 60%. Number of observations were: Gleason 6: 3, Gleason 7: 18, Gleason >7: 26. In this diagram, any tumor re-ocurrence, including those outside the IRE treatment field, was included. Despite this conservative approach, which included PCa in untreated volumes of the prostate, the recurrence rates for high-grade (Gleason >7) cancers fall inside the corridor of the recurrence rates after prostatectomy (obtained from the Han Tables of Johns-Hopkins) for comparable cancer stages and PSA-levels (blue and magenta dots, CI shown as bars for ease of perception).

**Fig 3:**
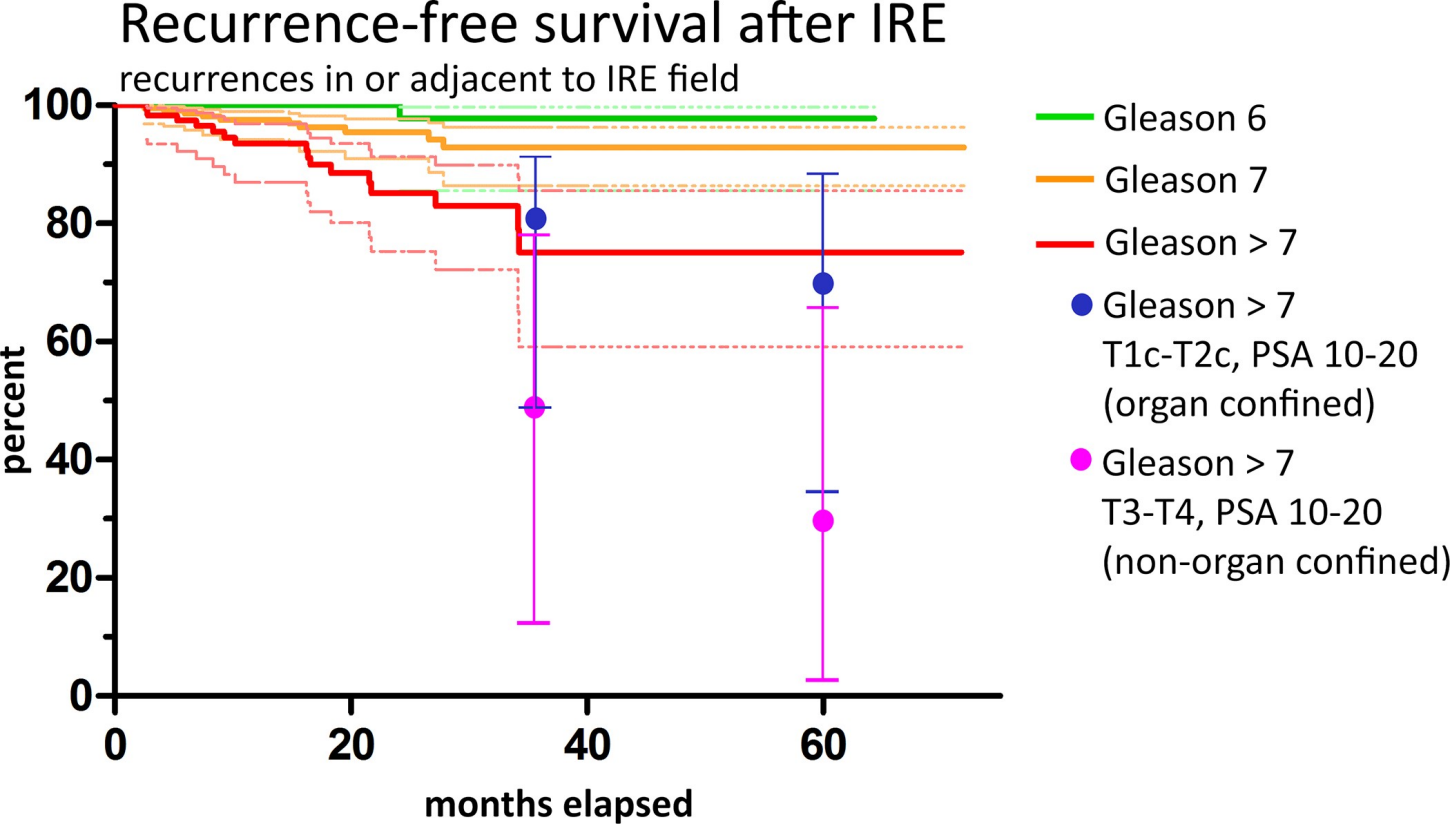
Kaplan-Meier curves for recurrent PCa for Gleason 6 (green line), Gleason 7 (orange line) and Gleason > 7 (red line) with CI of 95% (dashed thin lines in the associated colors), considering only the recurrences inside or at the margin of the IRE treatment field, excluding the 20 PCa which were located within residual prostate tissue and thus classified as out-of-field recurrence. Compared to the Kaplan-Meier curves in Fig 2, this analysis is more meaningful on the IRE technology specific aptitude for prostate cancer as it partly filters the diagnostic and focal therapy specific limitations. These were a total of 27 cases, with 1 patient Gleason 6, 10 patients Gleason 7, and 16 patients Gleason > 7. Here the recurrence rates after IRE are more similar to the recurrence rates after prostatectomy (values obtained from Han Tables [25], blue and magenta dots, CI shown as bars for ease of perception) for the lower stage cancers (organ confined disease, blue), even lower at 5-year follow-up: Gleason 6: 98% (64 months), Gleason 7: 93% (72 months), Gleason >7: 75% (72 months).

During the maximum follow-up period of 72 months, a total of 47 recurrent cancers (approx. 10%) were detected. The data is summarized in the Kaplan-Meier-diagram in Fig 2. As expected, most recurrences occurred in the high-grade and high-risk groups: 26 in the Gleason >7 group (red curve, 60% RFS at 72m), 18 in the Gleason 7 group (orange curve, 85% RFS at 72m). In the low-grade Gleason 6 group, 3 recurrences were observed (green curve, 94% RFS at 64m). It should be noted that all tumor re-occurrence, including those outside the IRE treatment field, were considered recurrences here. Only 27 of the 47 PCas which were classified as “recurrent” in Fig 2 occurred inside or at the margin of the IRE ablation zone, i.e. were “true” in-field recurrences; the other 20 cases occurred in untreated prostate tissue outside the IRE ablation zone (out-of-field recurrences), mainly in patients who had not had 3D-biopsy and/or endorectal mpMRI, i.e. in cases in which the diagnostic work-up was suboptimal. These should more appropriately be classified as diagnostic misses. The Kaplan-Meier-diagram based on cases with recurrent PCa in or adjacent to the treatment field is shown in Fig 3. Here, one patient had Gleason 6, 10 patients had Gleason 7a or 7b, and 16 patients had Gleason >7.

In order to investigate whether the calculated Kaplan-Meier predictions of survival proportions are not affected by follow-up times that are possibly too short, we selected all patients out of the 27 patients with recurrent disease in or adjacent to the IRE treatment field who had follow-up MRI/PSA of at least six months but no longer than 12 months before cut-off. The results are shown in Fig D in S1 Text. For this purpose, we selected 16 patients, 1 Gleason 6, 7 Gleason 7 and 8 patients Gleason >7. Their recurrence free survival proportions at 3 years were 95% (Gleason 6 green line), 90% (Gleason 7, orange line) and 53% (Gleason >7, red line).

Lastly, to better compare recurrence rates after IRE with those after RPE, we specifically selected patients with organ confined disease (Fig E in S1 Text) out of all the 47 recurrences (in-field and out-of-field recurrences). This resulted in 29 cases, with 2 Gleason 6, 15 Gleason 7 and 12 Gleason >7 patients. At 60 months follow-up, the survival proportions for IRE were: 95% for Gleason 6, 85% for Gleason 7 and 61% for Gleason >7.

## Discussion

This retrospective analysis of 471 treatments of PCa with IRE, comprising all clinical stages and recurrent disease, with over 6-year follow-up, to our knowledge constitutes as the largest patient cohort with the longest observation time. Our data appears coherent with the data previously published but extends to higher grades and stages confirming that IRE is an effective minimally-invasive procedure for the treatment of PCa.

The motivation to use IRE for the treatment of PCa was its promise of low invasiveness and toxicity, with the goal of preservation of vital anatomical structures surrounding the prostate, whose damage results in the urogenital functional deficits effected by surgery and radiation therapy.

Due to the individualized treatments that were provided, the data is heterogeneous. Ablation fields varied from focal to whole-gland, dependent on tumor stage, grade and distribution, and personal preferences (Table 2). This limits specific conclusions, but clearly proves the safety, scope and potential of IRE in a wide range of oncological manifestations.

Overall toxicity was low (Table 4), despite the fact that NVB, lower urinary sphincter (LUS), rectum, and the base of the bladder were often at least partially included in the IRE-field, mostly without lasting damage to these structures.

Most notable was the complete preservation of urinary continence after IRE in all patients, despite the partial or total inclusion of the LUS in the IRE-field. Urinary symptoms improved after IRE in patients with smaller IRE-fields, and slightly worsened for larger IRE-fields (Fig C Panel B in S1 Text). The effect is clearly time-dependent, as the majority of men with IPSS-Score elevation had decreased or disappearing symptoms after 12 months, on average even decreased IPSS-Scores (Fig C Panel A in S1 Text). This is due to the fact that most treatment fields reduced part of the benign cellular hyperplasia of the transitional zone of the prostate (BPH) but left the non-cellular elements (mainly fibers) *in situ*, which, when copious, can obstruct urinary outflow.

Occurrence of ED after IRE in this series was rare, but not zero. Standard evaluation of ED by IIEF-5 questionnaire revealed IRE-related severe ED in 11.3% of evaluated patients during the first year (transiently), and in 3% 12 months after IRE. As assessment of EF is difficult due to co-factors such as the nocebo effect [26], we also used our own subjective assessment (Table 3 and Fig 1D). The results confirm the transient nature of ED after IRE, with 45% of men experiencing transient ED (up to 12 months), but only 3% persistent severe ED, whilst 52% experiencing no ED at all.

Although frequently used, assessment of treatment toxicity on EF by standard criteria (i.e. severe ED defined by IIEF-5 <7) does not provide the full picture. Evaluation of the changes in the IIEF-5-Score as a function of time and ablation volume provides a more accurate picture. IRE-treatment induced a transient mean reduction of the IIEF-5-Score by 33%, and a longer-term (>12 months) reduction by 15%. Thus, there is regeneration of erectile function after IRE (Fig 1B, only the reduction in points plotted).

As to be expected, toxicity was clearly dependent on the size of the ablation zone and the extent to which the NVB were involved: the mean reduction of the IIEF-5-Score was twice as high in patients who had undergone whole-gland ablations (Fig 1A) as compared to smaller ablation fields. Again, the effect is time-dependent, with mean reduction of IIEF5-Score cutting half after 18 months (Fig 1B). Thus, there is regeneration after initial damage.

The correlation of toxicity with the size of the ablation zone is a complex matter. Especially in large (= more than 4 electrodes) ablation zones, effects like Joule heating and electrolysis overlap uncontrolled with the intended non-thermal IRE [20]. Larger treatment fields usually translate to higher probability of involvement of anatomically critical structures around the prostate which are important for urinary and erectile functions. Thermal IRE within these structures will most likely cause transient or permanent impairment of their functionality.

Initial local tumor control was achieved in all IRE-treated patients. Excluding planned two-stage treatments, recurrent rates of this patient cohort corresponds to an overall recurrence-free survival (RFS) rate of 90% (Fig 2). This is well within the acceptable range of ≥ 80% according to a consensus meeting on focal therapy [27], even though recurrences which were outside the treatment field were included and this cohort comprised many advanced high-risk cancers.

Comparison of the recurrence rates with those after RPE (Figs 2 and 3 and D and E in S1 Text; magenta and blue dots obtained from the Han Tables of Johns-Hopkins [25]) yielded similar results. For two reasons this should not be surprising: Firstly, local recurrences after RPE are frequent even without positive surgical margins [25], caused by cancer cells spread beyond the prostate. Secondly, IRE has been shown to reliably IRE kill all cells inside the treatment field [16]. Thus, RFS only partially depends on the treatment method and to a large extent on tumor biology in conjunction with the individual immune system [28]. There is, however, a bias: After RPE, biochemical recurrences might be identified earlier than when focal therapies are employed. Kaplan-Meier-curves presented here further showed a very similar slope as compared to the recently published data on 5-year outcomes of HIFU treatment for PCa [10], The fact that our Kaplan-Meier-curves presented here further have showed a very similar slopes as compared to the recently published data on 5-year outcomes of HIFU treatment for PCa [10] indicates the higher effectiveness of IRE for PCa treatment compared to HIFU, since the HIFU, even though their patient cohort comprised few high-risk and advanced disease patients, whilst our cohort included many of those. only included very few patients with advanced diseases and/or high Gleason Scores, whereas the results presented here have a high number of patients with high risk disease. Our data on outcome of patients with high-risk disease would thus be more comparable to [29], where Cryoablation was employed to perform whole gland procedures. After 5 years, the clinical recurrence free survival rate was reported to be 69%–again very similar to our results.

Being retrospective, the significance of our data is limited to heterogeneity, incompleteness and inconsistencies. One of our main concerns was that more patients with recurrences would be lost to follow-up than patients without, presumably because they were disappointed with IRE and turned towards RPE or RT. This might bias the data towards lower recurrence rates. The opposite would also be conceivable: Patients without signs of a recurrence might avoid follow-up because of inconvenience and cost. To test both hypothesis, we generated Kaplan-Meier-curves only including patients who fulfilled the follow-up requirements (MRI+PSA) for longer than one year and in whom the last complete follow-up was not older than one year (Fig 3). The curve suggests that within the confidence intervals there is no significant difference in the mean “slope”, hence the drop-out-rates appear to be evenly distributed between patients with and without recurrences.

Additionally, follow-up MRI and PSA scores had a median time till last follow-up data-point of 12m, with accessible follow-up data of less than a year in approximately half of all patients (Fig A in S1 Text). Fortunately, this fact does not result in a mean bias in the presented Kaplan-Meier-curves, as the drop out group due to follow-up seems randomly distributed. However, with PCa being a slowly growing cancer follow-up periods need to be extended to obtain a clearer understanding of oncological and functional outcomes.

In a clinical trial, re-biopsy would have been the standard follow-up method. In our series of individualized treatments, most patients refused re-biopsy, unless a recurrence could not be sufficiently ascertained by MRI and/or PSMA-PET/CT. Neither re-biopsy nor imaging are completely accurate: both might miss recurrences (false negatives), imaging might overestimate them (false positives, e.g. regenerative nodules and tracer accumulation in intra-prostatic urine collections). It is, however, unlikely, to repeatedly miss a PCa of clinically relevant size on MRI [9]. On the other hand, lesions below 5mm in size are unlikely to be missed by biopsies. We therefore concluded that MRI for small recurrences, and thus for recurrences in general, MRI would have lower specificity at equal or higher sensitivity than biopsy [30]. Thus, follow-up by PSA+MRI would be more likely to overestimate (false-positives) than underestimate (false-negatives) the rate of recurrences. This is supported by a recent study carried out by Scheltema et al. [21], which concludes that MRI is an accurate method to follow-up IRE-treatments.

## Conclusions

The retrospective evaluation of our data allows the conclusion that Irreversible Electroporation (IRE) is a safe, effective and suitable modality for the treatment of PCa at all clinical stages and recurrent disease. Treatment fields included the lower urinary sphincter, seminal vesicles, neurovascular bundles and in some cases partially the rectum and bladder, yet overall toxicity was low. Continence was preserved in all cases. In terms of local tumor control the comparison of IRE with radical prostatectomy (RPE) revealed similar recurrence rates over time, suggesting similar effectiveness of IRE to RPE. Thus the data illustrates the feasibility of IRE for PCa treatments, with similar functional outcomes and short to midterm efficacy to RPE, but much lower toxicity profile. Before IRE has the potential to become a new standard of care for the treatment of PCa, the presented data needs to be confirmed by more systematic studies. A more stringent evaluation is of course required, of which the multi-center registry (NCT02255890) is a first step, as well as optimization and standardization of diagnostic work-up, patient selection, the technical procedure of IRE and follow-up regime.

## Supporting information

S1 Text Text, containing additional information and figures on technical and methodical details as referenced in the manuscript. (PDF)
