# Supplementary material for: Prostate cancer treatment with Irreversible Electroporation (IRE): Safety, efficacy and clinical experience in 471 treatments
Source: PLoS One. 2019 Apr 15;14(4):e0215093. doi: 10.1371/journal.pone.0215093 (PMC6464181; doi:10.1371/journal.pone.0215093)
Supplement: S1 Text — (PDF) [file pone.0215093.s001.pdf]

Supporting Information to: Prostate Cancer Treatment with Irreversible Electroporation (IRE): Safety, Efficacy and Clinical Experience in 471 Treatments

Diagnostic Work-up

Magnetic Resonance Imaging (MRI)

MRI was carried out on a 1.5 Tesla scanner (Siemens Avanto) with the following sequences (VS = voxel size): T2 ax whole-pelvis TSE (VS 1.3x1.0x5.5mm^3); T2 ax, sag, cor small FOV (VS 0.7x0.6x3.0mm^3); T1 small FOV (voxel size 1.2x0.8x3.0mm^3). VIBE (DCE) (VA 1.7x1.3x3.0mm^3, 20-measurments, each 8sec; EPI (diffusion) small FOV (VS 1.5x1.5x3.5mm^3, b=0, 50, 150, 500, 800, 1200 s/mm^2; ADC and b2000 were extrapolated, for ADC a mono-exponential fit was employed; Dixon-VIBE whole-pelvis sequence (VS 1.1x1.1x1.3mm^3), in some cases 1H-MRS using chemical shift imaging on a prostate only FOV. Most scans for staging and planning before IRE were carried out with an endo-rectal coil (MEDRAD eCoil 1.5). In some cases, external MRI with different parameters were used if they were deemed of acceptable quality and the patient refused a repeat MRI. 24h post-procedure MRI was optimized for treatment zone delineation (VIBE DCE and Dixon-VIBE, v.s.), and exclusion of hemorrhage and organ damage (rectum, bladder, etc.). This protocol comprised the same sequences but with adjustments to the use without an endo-rectal coil. Most follow-up MRIs, particularly those for exclusion of recurrences were performed with an endo-rectal coil employing the same protocol. The evaluation was performed in accordance to PI-RADS v1 and v2 until and after January 2016, respectively.

3D-Mapping Biopsy

The procedure was carried out under full anesthesia (TIVA) in lithotomy position with with a stepper and brachytherapy grid (CIVICO medical solutions) placed in front of the perineum under US (BK

Medical Flex Focus 300 or BK3000) guidance. Biopsies (Uromed Kurt Drews KG) of 15 or 22 mm length were taken through the perineum every 5 mm (spacing of the grid) inside the prostate. The matrix was a 5mm spaced brachygrid for X and Y axis orientation. X, y and z-coordinates of each biopsy were recorded, affording a color-coded (for Gleason-score) 3D-histopathological model to be reconstructed from the results using a custom programmed computer software, with US and MRI cut-plane overlays. When deemed necessary, additional cognitive fusion biopsies were obtained.

Follow-up

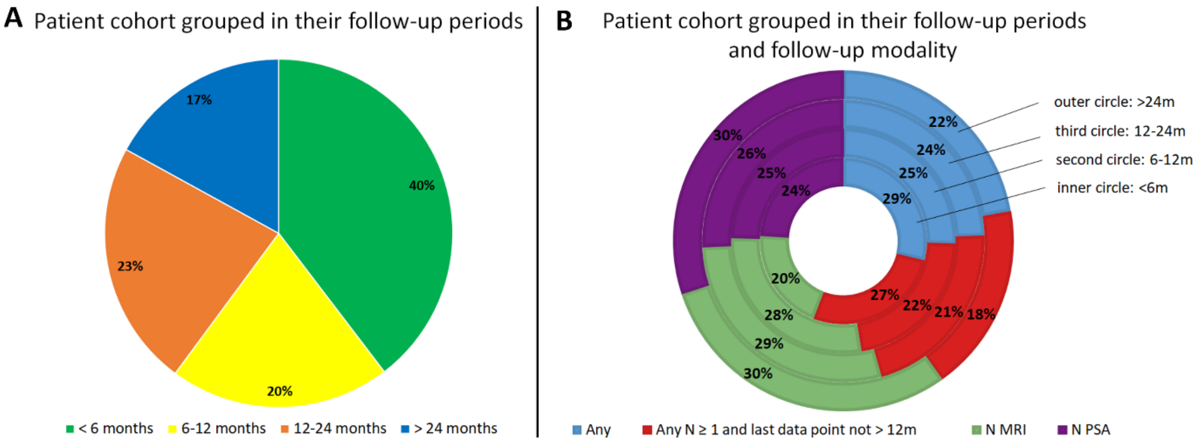

Fig A: Patient cohort grouped according to their most recent follow-up data point (MRI or PSA). Left: green = last data point acquired less than 6 months after IRE, yellow = 6 to 12 months, orange = one to two years, blue over 2 years after IRE. **Right:** Patients grouped in most recent follow-up periods and follow-up modality. Inner circle: last data point acquired less than 6 months after IRE, second circle: 6 to 12 months, third circle one to two years, outer circle over 2 years of follow-up post IRE. Colors indicate follow-up modality; green MRI, purple PSA, blue either MRI or PSA, red more than one follow-up data point and last data point not over 12 months prior cut-off.

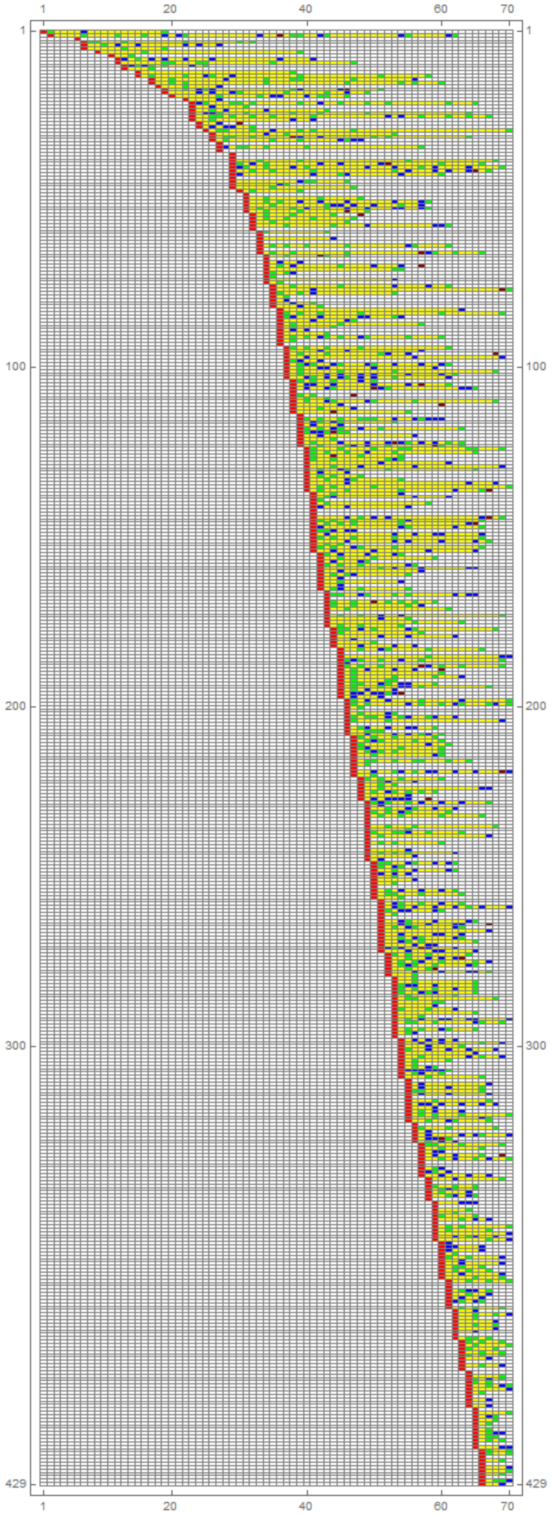

Fig B: Follow-up duration matrix of all 429 patients. X-Axis: month since first IRE. Y-Axis: patient number. Red: time of IRE; green: on-site MRI and PSA follow-up on site, blue: External MRI or PSA; dark-red: recurrence found; yellow: follow-up time (see discussion section).

Urinary Continence

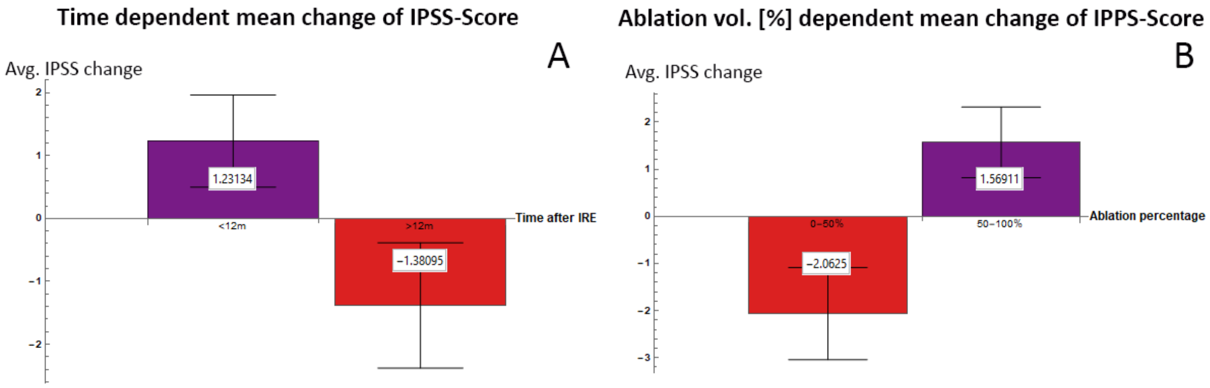

Fig C: Summary of outcome on IPSS-Scores after IRE treatment of the prostate. A. Time-dependent mean change of IPSS-Scores: less than 12 months after IRE (left, purple, 1.23 points increase) and over 12 months post IRE (right, red, 1.38 points decrease). **B.** Ablation volume dependent mean reduction of IPSS-Score grouped into patients who had an ablation volume of 50% or less (left, purple, 2.06 points decrease) or more than 50% of prostate tissue treated (right, red, 1.57 points increase). **IPSS-Score Interpretation:** IPSS has a maximum of 35 points, with symptom score ≤ 7 classified as mild, and > 20 as severe.

155 patients had a valid IPSS questionnaire filled out before and after IRE (the median time of the first follow-up questionnaire was 101 days after IRE), 82 had at least three questionnaires filled out with the latest one dated ≥ 12 months post treatment. The average value of the first IPSS-Score within the first 12 months after IRE is slightly higher (1.23 points (StdDev 8.45 StdErr 0.732)) than baseline (Fig A, Panel A, purple, left), with 12 of the 155 cases (7.7%) temporarily increasing to scores from below 8 to above 19 (severe symptoms). In one of the 82 cases with at least three follow-up IPSS-Scores (1.2%) the last available data point still shows a score above 20. On average, the IPSS-Score is lower than the initial score ≥ 12 months after IRE (-1.38 points (S1 Fig, Panel A, red, right, StdDev 4.57 StdErr 1.00)). The Man-Whitney P-Value between the mean score differences within and

after 12 months relative to the IRE date is p=0.191. 125 patients had validly filled out the question on quality of life relating urinary symptoms before and after IRE. 91 patients (72.8%) reported a better or equal quality of life in terms of urinary symptoms and 34 (27.2%) report a decrease. To the date of data acquisition cutoff, in one case, the last available data point still shows dissatisfaction (≥ 5 points) in a patient who was initially satisfied (≤ 2 points). S1 Fig, Panel B shows the dependency of ablation volume in relation to the IPSS-Score: A reduction of the average IPSS-Score can be seen in cases of focal ablation (-2.06 points, left, purple, StdDev 5.53, StdErr 0.98), whereas there is a slight increase in patients who had over 50% of their prostate ablated (+1.57 points, right, red, StdDev 8.31, StdErr 0.75). Additionally, positive correlation between focal (ablation volume <50%) and whole gland (ablation volume >90%) in terms of IPSS Score was observed (Mann-Whitney p=0.0027).

Efficacy – Recurrence free survival

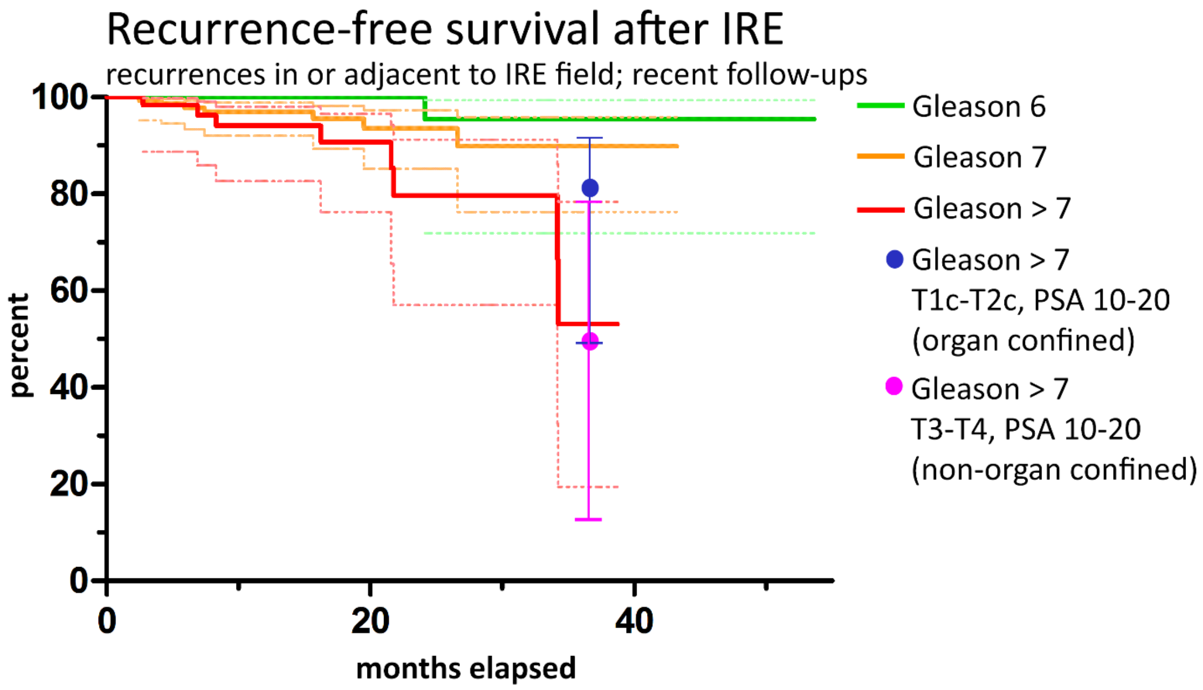

Fig D: RFS in cases of recurrences in or adjacent to IRE field and with recent follow-up. Kaplan-Meier curves for recurrent PCa for Gleason 6 (green line), Gleason 7 (orange line) and Gleason > 7 (red line) with CI of 95% (dashed thin lines in the associated colors), considering only patients with MRI+PSA on-site follow-up of at least 6 months but not longer than 12 months before cut-off. One patient had Gleason 6, and 7 and 8 patients had Gleason 7 and > 7, respectively. At 3 years, survival proportions were: Gleason 6: 95%, Gleason 7: 90%, Gleason >7: 53%. Values compared to RPE obtained from Han Tables [1] (blue and magenta dots, CI shown as bars for ease of perception).

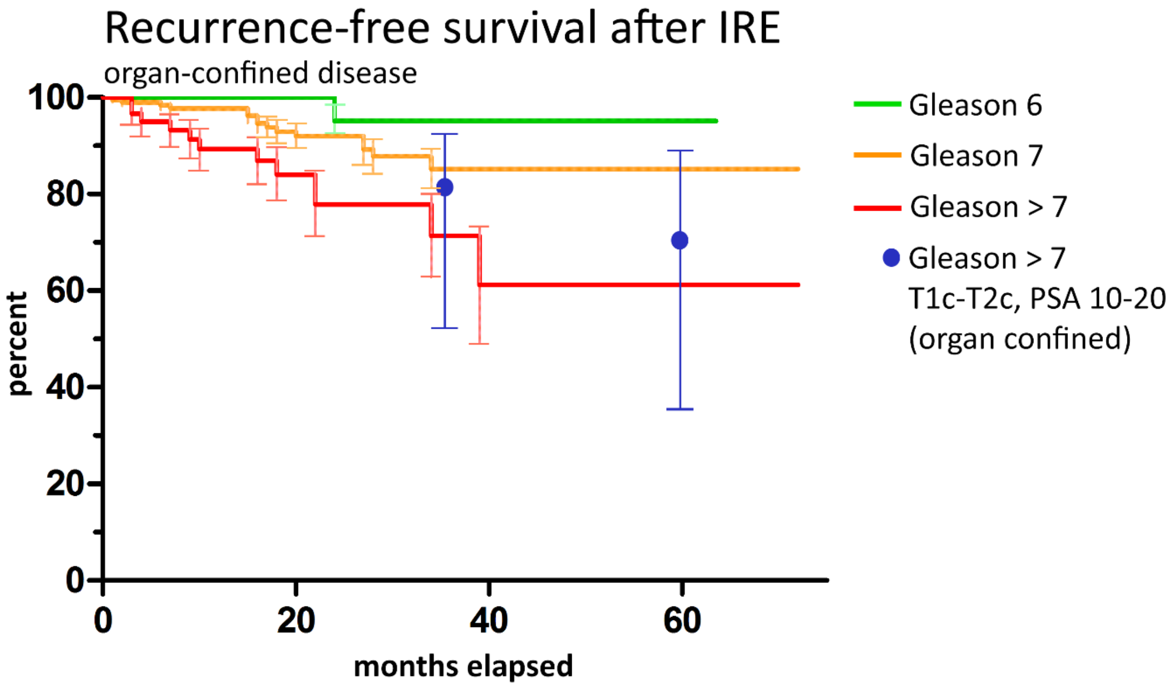

Fig E: RFS in organ-confined disease. Kaplan-Meier curves for recurrent PCa for Gleason 6 (green line), Gleason 7 (orange line) and Gleason > 7 (red line) with standard error (error bars in the associated colors), considering only patients with organ-confined disease. Number of observations: Gleason 6: 2, Gleason 7: 15, Gleason >7: 12. We specifically compared Gleason >7 organ-confined disease to recurrence rates after prostatectomy (values obtained from Han Tables [1]) for the same patient group (blue dots with CI shown as bar for ease of perception). At 60 months, the recurrence-free survival rates compare (IRE: 61% (73-49%), prostatectomy: 71% (81-35%)).

References

1. Han, M., Partin, A.W., Zahurak, M, Piantadosi, S., Epstein, J.I., Walsh P.C. Biochemical (Prostate Specific Antigen) Recurrence Probability Following Radical Prostatectomy for Clinically Localized Prostate Cancer. J Urol. 2003;169(2):517–23. doi: 10.1016/S0022-5347(05)63946-8.
